# Paris MEM: a study protocol for an effectiveness and efficiency trial on the treatment of traumatic stress in France after the 2015–16 terrorist attacks

**DOI:** 10.1186/s12888-019-2283-4

**Published:** 2019-11-08

**Authors:** A. Brunet, A. Ayrolles, L. Gambotti, R. Maatoug, C. Estellat, M. Descamps, N. Girault, K. Kalalou, G. Abgrall, F. Ducrocq, G. Vaiva, N. Jaafari, M. O. Krebs, E. Castaigne, I. Hanafy, M. Benoit, S. Mouchabac, M. C. Cabié, O. Guillin, F. Hodeib, I. Durand-Zaleski, B. Millet

**Affiliations:** 1 Département de Psychiatrie adulte, boulevard de l’Hôpital, 75013 Paris, France; 2 Department of Psychiatry, McGill University, Montréal, Canada; 3 Département de Psychiatrie adulte, Hôpital Universitaire de la Pitié Salpêtrière, Assistance Publique - Hôpitaux de Paris, boulevard de l’Hôpital, 75013 Paris, France; 4 Unité de recherche clinique, EPS de Ville Evrard, G03, 5 rue du Dr Delafontaine, 93200 Saint-Denis, France; 5 Assistance Publique - Hôpitaux de Paris -, Hôtel-Dieu, 75004 Paris, France; 6 France CHRU de Lille, Pôle de Psychiatrie Médecine Légale et Santé en milieu Pénitentiaire, SCA-Lab CNRS UMR 9193, 59037 cedex Lille, France; 7 CIC INSERM U802, CHU de Poitiers, Unité de recherche clinique intersectorielle en psychiatrie du Centre Hospitalier Henri Laborit, 86022 Poitiers, France; 8 Centre Hospitalier Sainte Anne, Service Hospitalo-Universitaire, Faculté de Médecine Paris Descartes, Université Paris Descartes, Paris, France; 9 Service de Psychiatrie, CHU de Bicêtre, HUPS, APHP 78, rue du général Leclerc, 94270 Le Kremlin Bicêtre, France; 10 CH Marne La Vallée, Service de Médecine Légale, 77420 Marne-La-Vallée, France; 11 Clinical Neuroscience Department Hospital Pasteur 1, France University of Côte d’Azur, 30 avenue de la voie, 06002 NICE cedex 1 Romaine, France; 12 Département de psychiatrie et de psychologie médicale de l’adulte, Hôpital universitaire Saint-Antoine, Université Pierre et Marie Curie, Paris VI - AP-HP, 184 rue du Faubourg-Saint-Antoine, 75012 Paris, France; 13 Pôle Paris 11 Les Hôpitaux de Saint Maurice, 12-14 rue Val d’Osne, 94410 St Maurice, France; 14 Service Hospitalo-universitaire, CH du Rouvray, 4 rue Paul Eluard, 76300 Sotteville-lès-Rouen, France; 15 unité Inserm U1079 Faculté de médecine et de pharmacie, 76000 Rouen, France; 16 ECEVE, UMR 1123 URCEco Ile de France Hôtel Dieu, 1 place du Parvis de Notre Dame, 75004 Paris, France

**Keywords:** Post-traumatic stress disorder (PTSD), Terrorism, Reconsolidation, Cost-effectiveness trial

## Abstract

**Background:**

The Paris and Nice terrorist attacks affected a thousand of trauma victims and first-line responders. Because there were concerns that this might represent the first of several attacks, there was a need to quickly enhance the local capacities to treat a large number of individuals suffering from trauma-related disorders. Since Reconsolidation Therapy (RT) is brief, relatively easy to learn, well tolerated and effective, it appeared as the ideal first-line treatment to teach to clinicians in this context.

**Methods:**

This study protocol is a two-arm non-randomized, multicenter controlled trial, comparing RT to treatment as usual for the treatment of trauma-related disorders. RT consists of actively recalling one’s traumatic event under the influence of the ß-blocker propranolol, once a week, for 10–25 min with a therapist, over 6 consecutive weeks. This protocol evaluates the feasibility, effectiveness, and cost-utility of implementing RT as part of a large multi-center (*N* = 400) pragmatic trial with a one-year follow-up.

**Discussion:**

Paris MEM is the largest trial to date assessing the efficiency of RT in the aftermath of a large-scale man-made disaster. RT could possibly reinforce the therapeutic arsenal for the treatment of patients suffering from trauma-related disorders, not only for communities in western countries but also worldwide for terror- or disaster-stricken communities.

**Trial registration:**

Clinical Trials (ClinicalTrials.gov). June 3, 2016. NCT02789982.

## Background

The terrorist attacks that struck France in 2015 and 2016 were the most devastating and deadly events to occur on its territory since WWII, with a thousand of individuals physically or psychologically injured by the attacks according to official numbers [1]. Past research suggest that up to 20% of individuals exposed to this type of event develop posttraumatic stress disorder (PTSD) [2]. PTSD’s core symptoms are: re-experiencing, avoidance, alterations in mood and cognition, and hyperarousal [3]. Epidemiological studies indicate a median time of 3 years before remission when the disorder is treated, and of 5 years when left untreated, with a significant proportion of unremitted individuals 10 years after trauma exposure [2]. PTSD is also frequently comorbid with major depression (48–55%), dysthymia (21–23%), generalized anxiety disorder (15–17%), social phobia (28%), specific phobia (29–31%), panic disorder (7–13%), alcohol abuse (28–52%), and substance abuse (27–35%) [2, 4, 5]. Given the magnitude of the events that took place in France in 2015–16, and considering the poor prognosis of PTSD from a human suffering perspective, in the aftermath of the terrorist attacks there was an urgent need to quickly enhance the treatment resources in France. There were also fears that this attack might be the first one of a series of such events. We also reasoned that this massive terrorist attack might serve as a learning experience and, if successful, the treatment strategy deployed in Paris and Nice might serve in similar circumstances elsewhere in the future.

In order to address this unexpected plight, the research center of the Douglas Mental Health University Institute (Canada) and the public healthcare system of Paris (*Assistance Publique – Hôpitaux de Paris [AP-HP], University Hospitals of Ile de France, France*) worked together to build an innovative care proposition, named Paris MEM (shorthand for ‘*Paris, Mémoire Vive’*). The aims of this project were: (a) to quickly train a group of at least 40 mental health professionals on 20 sites in using Reconsolidation Therapy (RT), as developed by Alain Brunet [6]; (b) to offer to patients, in addition to the already existing care propositions (i.e., treatment as usual), a brief innovative first line treatment for PTSD; (c) to assess the effectiveness and the cost-utility of RT versus treatment as usual (TAU), and (d) to enhance the overall preparedness in France, in the event of subsequent large scale terrorist attacks. In this context, RT appeared as an appealing treatment modality since it is brief, well tolerated and effective [7] and its implementation seemed relatively easy.

### Reconsolidation as an emerging therapy for PTSD

Upon retrieval, under conditions of mismatch, a consolidated memory returns to a labile state before undergoing *re*-consolidation [8–10]. A large body of animal and human research shows that a deconsolidated memory can be impaired after its reactivation using a pharmacological agent (for a review see [11, 12]), notably with the β-adrenergic receptor blocker propranolol (for a review see [13, 14]). Propranolol is a lipophilic β-blocker, widely used for a variety of diseases in the last 50 years, with very few contraindications and side effects.

In 2008, Brunet et al. [15] were the first to show in a randomized controlled trial that long-standing traumatic memories can be durably toned down, using a treatment protocol congruent with reconsolidation theory. This intervention protocol was further refined and tested in three independent open-label trials in France, Canada and the United States [6]. The results showed a significant posttreatment decrease of PTSD symptoms, as well as at the 6-month follow-up. A recent randomized controlled trial comparing RT versus trauma reactivation + placebo confirmed such findings [7]. Reconsolidation impairment using propranolol has also been used with success in small randomized protocols for the treatment of phobias [16] and addiction [17]. In its current form, the treatment protocol for PTSD requires six once-a-week sessions of 10–25 min each with a therapist.

### Current treatments for PTSD

Psychotherapies such as Cognitive-Behavioral Therapy (CBT) and Eye Movement Desensitization and Reprocessing (EMDR), and pharmacological interventions such as the Selective Serotonin Reuptake Inhibitors (SSRIs) are all among the recommended treatment modalities for PTSD according to most guidelines [14, 18–21] and according to a Cochrane review [22]. In three randomized controlled trials (*N* = 1070), paroxetine had a significantly higher response rate than placebo ranging from 29 to 67% after 12 weeks [23–25]. The mean time for response ranged between 4 and 6 weeks. SSRIs such as paroxetine, while effective in the treatment of PTSD do not, however, offer a cure for PTSD. They must be taken daily for at least 1 year, and their side effects often lead patients to discontinue them [26]. CBT, including EMDR, comprise a variety of related approaches that have at their core exposure and/or cognitive restructuring as their main therapeutic ingredient [27, 28]. The recommended duration of individual therapy is 6–12 ninety-minutes sessions of treatment, offered once or twice weekly in an outpatient setting [14, 27]. CBT and EMDR both require strong educational and clinical backgrounds and can hardly be quickly learned in the aftermath of mass trauma. Furthermore, of the 2/3 of those who benefit from CBT at post-treatment, half of them relapse within 1 year, thereby reducing the treatment’s efficiency [29]. For all those reasons, we opted to teach RT therapy to the clinicians participating in this trial.

### Efficacy, effectiveness and efficiency

As summarized by Kim [30], efficacy is the extent to which an intervention works under ideal circumstances while effectiveness assesses whether an intervention works when provided under usual circumstances of healthcare practice. Research on effectiveness is also described as pragmatic trials (see Additional file 1) [31]. Pragmatic trials have several characteristics: 1) patient eligibility and recruitment should reflect routine practice; settings should reflect the care facilities available to a diverse population; 2) the intervention should be provided by normal staff with routine training; 3) intensity of follow-up should be similar to typical follow-up in usual care; 4) primary outcome should be directly relevant to participants and all patients should be included in the analysis of the primary outcome; 5) outcome measures are patient-centered and incorporate broad measures of health. Finally, efficiency refers to the ability to achieve a therapeutic goal using a lesser amount of time and resources than what is typically observed. Typically, efficiency is assessed by cost-utility analysis.

The co-primary aims of this pragmatic trial are to evaluate the effectiveness and the efficiency of RT therapy, as deployed in a post-terrorist attack real-world setting. Specifically, we wish to explore the feasibility of implementing in routine practice an innovative treatment for PTSD on a large scale and in a short period of time; to evaluate the acceptability of the treatment by the clinicians and from the patients’ perspectives; to assess the efficacy of RT; to determine its cost-utility compared to treatment as usual (TAU) over a 1-year period.

## Methods

### Study design

This study is a two-arm non-randomized, multicenter controlled trial, comparing RT to TAU for the treatment of trauma-related disorders. As shown in Table 1, psychometric assessments are made at baseline, after 6 weeks of treatment, as well as 3 and 12 months following study inclusion. In order to enhance external validity this study protocol is implemented into the usual healthcare system in France. Patients are offered the choice of the intervention they wish to receive. The study protocol was approved by local ethics committee (*Comité de protection des personnes* for Ile de France VI #CPP/14–16), and regulatory agencies (*Commission nationale de l’informatique et des libertés* #GPR1713292F and *Agence nationale de sécurité du medicament.* We plan to include up to 400 participants with the possibility of stopping at the end of the pre-specified inclusion period if at least 200 receiving RT are enrolled.

**Table 1:** Overview of measures and time of assessment

Instrument	Variable	T0	Treatment sessions						T1	T2	T3
			t1	t2	t3	t4	t5	t6			
Demographics	*Sociodemographic characteristics*	X									
PCL-S	*PTSD symptom severity*	X	x	x	x	x	x	x	X	X	X
CGI	*Global patients’ condition*	*	x	x	x	x	x	x	*	*	*
MINI-S	*Diagnosis and comorbidities*	X							X	X	X
PDI	*Severity of trauma exposure*	X									
PDEQ	*Peritraumatic dissociation*	X									
WHOQOL-BREF	*Quality of life*	X							X	X	X
EQ-5D-5 L	*Quality of life*	X							X	X	X
MEDEC	*Health service utilization*	X							X	X	X
HSCL-25	*Anxiety and depressive symptoms*	X							X	X	X
QFS	*Social functioning*	X							X	X	X

*Assessed by a clinician blind to the patient’s treatment modality

T0: baseline assessment

T1: posttreatment assessment (week 7)

T2: 3-month follow-up assessment

T3: one-year follow-up assessment

PCL-S: PTSD Checklist – Specific Version, CGI: Clinical Global Impression, MINI-S: Mini International Neuropsychiatric Interview for DSM-5, PDI: Peritraumatic Distress Inventory, PDEQ: Peritraumatic Dissociative Experiences Questionnaire, WHOQOL-BREF: World Health Organization Quality of Life (brief), EQ-5D-5 L: EuroQol Questionnaire, MEDEC: Medico-Economic Questionnaire, HSCL-25: Hopkins Symptom Checklist, QFS: Social Functioning Questionnaire

### Eligibility criteria

**Fig. 1:**
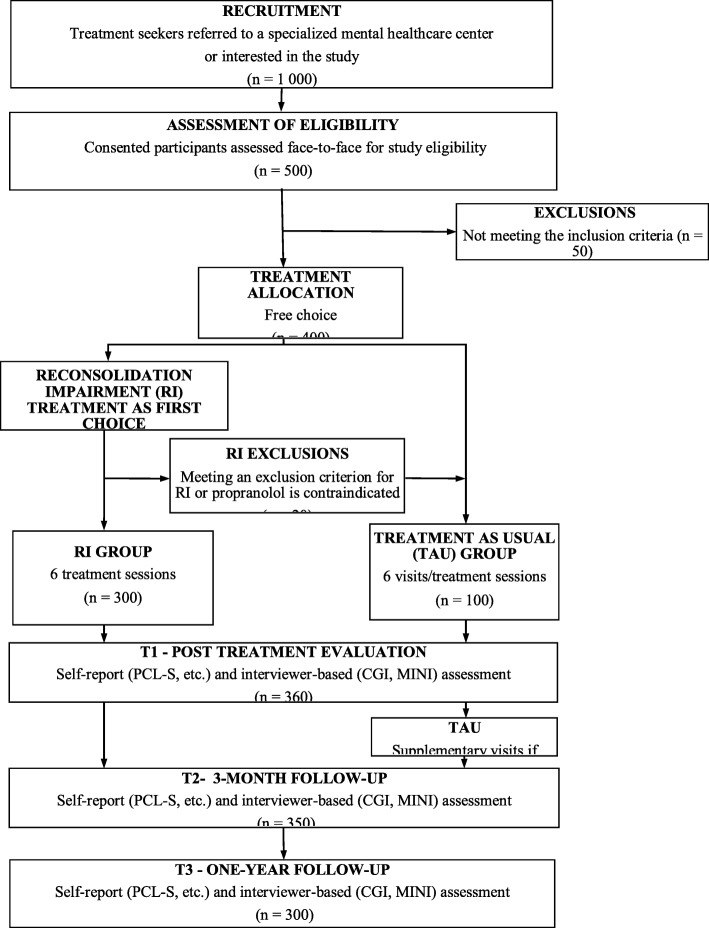
Study flowchart

Participants will be at least 18 years old without any upper age limit. Eligible participants must read and speak French, have a DSM-5 diagnosis of PTSD, adjustment disorder or any other specified trauma-related disorder. They must have a symptom score above 43 on the PTSD Checklist [32] and be at least moderately ill according to the Clinical Global Impression scale. They must not have a severe mental or neurological disorder (i.e., a history of bipolar or psychotic disorder, acute suicidal, homicidal, or self-injuring intentions, a traumatic brain injury in the last 5 years, an alcohol or substance dependence disorder), nor be involved in trauma-related litigation. Pregnant or breastfeeding women cannot participate in the trial for security reasons. In addition, to participate in the RT group, patients taking psychotropic medications must be on a stable dosage for at least 2 months, they must not use any medications that involve a dangerous interaction with propranolol, or have a resting heart rate inferior to 55 beats per minute, or a resting systolic blood pressure below 100 mmHg; any other contraindicating medical condition, as determined by the study physician (Fig. 1).

### Procedure

Patients presenting with trauma-related symptoms to participating mental healthcare centers in Paris (15 centers), Lille, Fort-de-France, Poitiers and Nice (2 centers) regions are informed of the study. After the study has been fully explained to them, interested patients are asked to sign the informed consent form. The clinician then formally investigates if the study requirements are fulfilled and determines patient eligibility. Based on the patients’ choice and their eligibility for the RT group, enrolled patients are then allocated to RT or TAU. The treatment begins within 7 days of enrolment. A series of six treatment sessions are set on a once a week basis, but with a margin of 4–15 days between sessions. The patients’ general practitioner is informed about their patient’s participation in the study. The symptom assessments are conducted for both groups at baseline, and then 7, 13, and 52 weeks after study inclusion.

### Clinicians training

A two-day course is provided to the participating clinicians by the first author (A.B.). Clinicians are either medical doctors with a mental health background or practice, practicing residents and psychiatrists, or practicing psychologists. The first part of the training involves the proper identification of trauma-related disorders. The second part reviews the empirical evidence in favor of RI. The third part is practical and addresses propranolol prescription rules, role playing, RT procedures (see also Brunet et al., 2018) and research protocol information. Online clinical supervision is offered to each clinician via a private forum moderated by A.B. as well as personalized telephone support with A.B. or B.M. regarding specific questions, on an as needed basis. Treatment adherence is monitored in the following way: selection of the index traumatic event, performance of a cardiogram (first session only), oral propranolol dosage relative to ideal body mass, ingestion of a light snack with the propranolol medication, heart rate and blood pressure at baseline and, following ingestion of propranolol, the participant’s side effects to propranolol, the time elapsed between propranolol ingestion and the beginning of the treatment session, the completion of a self-report PTSD symptom measure by the patient covering the previous week, the length (number of pages) of the trauma narrative, use of the first-person singular, and verb tense (present) of the trauma narrative, the inclusion in the trauma narrative of five or more descriptors (from a list) of peritraumatic physical reaction, whether the patient actually read aloud his or her full script, the designation to the therapist of the *hot spot* in the trauma narrative, the possibility to revise/supplement the trauma narrative at each treatment visit, the number (six) and duration (10–25 min.) of the treatment visits, the number of days between each treatment visit, as well as the lack of use of any other empirically-validated treatment method for PTSD by the treating clinician during the treatment sessions.

### Study treatments

Reconsolidation Therapy™ (RT) consists in actively recalling one’s *index* (i.e. worst/traumatic) event, using a specific memory reactivation protocol, under the influence of the ß-blocker propranolol, once a week, for 10–25 min with a therapist, over 6 consecutive weeks. More specifically, 1 mg/kg of oral propranolol (Inderal™/Avlocardyl™) based on ideal body mass index (BMI) is given at the treatment center 75 min (+/− 15 min) before the session. Once the session begins, the patients are asked to write (session 1) and read out-loud (sessions 1–6) a 1–2 pages narrative of their traumatic event. On subsequent sessions, the patients are asked if they wish to update or modify their narrative to include new/more detailed information thatg bothered them during the previous week. The trauma narrative, a description of the event and of the trauma’s *hot spot* (i.e. the most bothersome/unacceptable part of the event), includes at least five bodily sensations and is written in the first person singular, present tense [15].

Treatment as usual (TAU) involves receiving the PTSD treatment offered locally at this treatment center, whatever this treatment may be. In most centers, TAU was an SSRI (most commonly, paroxetine 20–60 mg started at the first treatment visit), and/or a form of psychotherapy, typically CBT, EMDR, supportive therapy or psychodynamic therapy [26–28].

### Instruments

The primary outcome for the effectiveness analysis is the difference between the group mean PTSD Checklist-Specific (PCL-S) score at inclusion (T0) minus the same score obtained at the one-year follow-up (T3). The PCL-S will be administered at baseline (T0), at each treatment session (t1-t6) and 1 week posttreatment (T1), 3-month follow-up after baseline (T2) and one-year follow-up after baseline (T3).

**Fig. 2:**
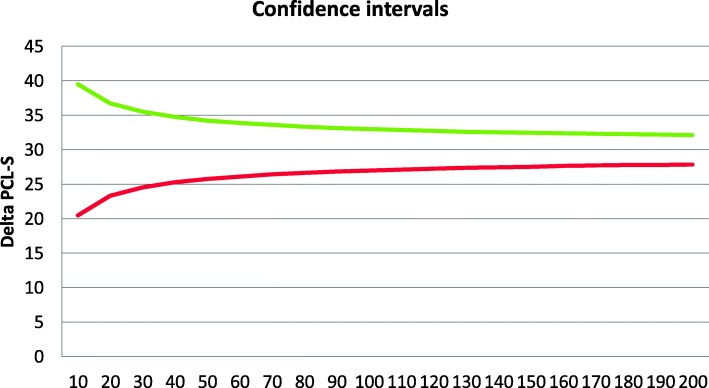
Evolution of the confidence interval according to the sample size and the delta PCL-S (green line: delta PCL-S above 30; red line: delta PCL-S below 30)

The reliable and valid PCL-S [32, 33] is a 17-item self-report scale that assesses DSM-IV-TR PTSD symptoms in the past week from the perspective of the patient. (No validated French PCL version compatible with the DSM-5 was available when the study began). The PCL-S ranges between 17 (no symptom) and 85 (maximum score). For study inclusion, a cut-off score of equal or greater than 44 is used, as done by others [34, 35] (Fig. 2).

The Clinical Global Impression (CGI) scale is used as a global evaluation of the patients’ condition and symptom evolution. The CGI is composed of two parts: illness severity (CGI-S) and improvement (CGI-I), each of which is rated on a 7-point scale where 1 represents health (not ill/very much improved) and 7 represents illness (extremely ill/much worse). The CGI is administered by a clinician blind to the patient’s treatment modality.

*PTSD or adjustment disorder diagnosis and comorbidities*, are assessed with the MINI International Neuropsychiatric Interview (MINI-S), a structured diagnostic interview based on the DSM-5. PTSD, adjustment disorder, anxiety modules as well as lifetime and current depression, suicidality, obsessive-compulsive disorder, alcohol and substance abuse and dependence are assessed in order to report any comorbid disorders that are frequently associated with PTSD. The MINI-S is the new version of the MINI interview [36]; its validation in French is in progress at our center and elsewhere.

*Distress and dissociation at the time of trauma* are assessed with the reliable and valid Peritraumatic Distress Inventory (PDI) [37, 38] and the Peritraumatic Dissociative Experiences Questionnaire (PDEQ) [39, 40], respectively. The 13-item self-report PDI documents the recalled emotional responses experienced at the time of the trauma and, as such indexes the subjective severity of the trauma from the patient’s perspective. It ranges between 0 (no symptom) and 52 [41]. The 10-item self-report PDEQ measures the recalled dissociative reactions experienced during or immediately after the traumatic event and is an index of poor prognosis. Items are scored from 1 to 5 with a total score ranging between 10 (no symptom) and 50.

The primary outcome for efficiency (cost-utility) analysis is *Quality of life* at 12 months assessed with the reliable and valid EuroQol questionnaire (EQ-5D-5 L) [42, 43]. The EQ-5D-5 L assesses five health dimensions: mobility, self-care, usual activities, pain/discomfort and anxiety/depression. Each dimension is rated on five levels of severity [42, 43]. The utility value set for each score is available for the French population. These values will be used in the assessment of efficiency of RT. We will also use the 26-item self-report World Health Organization Quality of Life WHOQOL-Bref [44, 45], which measures the extent to which illness impairs the subjective wellbeing of patients at the time of the assessment. Each item scored 1–5 is summed and transformed into a 0 (poorest QOL) to 100 (best QOL) symptom score. The scale produces four main scores: physical, psychological, social relationship and environment.

*Costs related to healthcare utilization and work status* are evaluated with the MEDico-EConomic Questionnaire (MEDEC) adapted from the Client Service Receipt Inventory [46, 47] in order to meet the specificity of the French health system. This inventory collects retrospective information on health service utilisation in order to estimate illness related costs. It includes a history of hospitalization, sick leave, treatments received, medical consultations, as well as all paramedic consultations regardless of the reason.

This economic evaluation is conducted in accordance with the recommended methods of the HAS (*Haute Autorité de Santé* or French Health Authority) and the CHEERS (Consolidated Health Economic Evaluation Reporting Standards) [48].

*Social functioning* is evaluated with the Social Functioning Scale (QFS) [49]. The 16-item self-report QFS assesses the frequency and satisfaction with a number of social behaviours in a psychiatric adult population. Each item is scored from 1 to 5 with frequency and satisfaction scales ranges between 8 and 40 and a total range of 16 to 80 over the previous 2 weeks and is sensitive to change after treatment [49].

*General anxiety and depressive symptoms* are evaluated using the 25-item self-report Hopkins Symptom Checklist (HSCL-25) [50], which includes a subscale measuring anxiety and another measuring depression (each item is scored 1–4, and the total mean score also ranges from 1 to 4, with the clinical cut-off being set at 1,75). The total score is correlated with emotional distress of unspecified diagnosis, and the depression score is correlated with major depression as defined by the DSM-IV-TR [51].

### Statistical analyses

The clinical scores will be analyzed using an intention to treat (ITT) analysis: all patients allocated to RT or TAU will be analyzed. The collected data will be used to describe the 2 cohorts: the RT group and the TAU group. Variables will comprise socio-demographic parameters, duration of PTSD, psychiatric comorbidities, pre-morbid disorders, and co-prescriptions. As described above, the primary outcome for effectiveness analysis is based on the delta (T3 minus T0) of PTSD *symptom severity* (PCL-S). A first indicator will be the mean PCL- S difference between T3 and T0. The delta of EQ5D-5 L (T3 minus T0) examines efficiency from the health economic perspective.

A regression model will be used to calculate a second indicator based on the PCL-S values (t1-t6) collected between T0 and T1, allowing for the evaluation of PTSD symptoms evolution over time and its variation between individuals. A third indicator will be the rate of patients considered as treatment responders (PCL-S score < 44) at Week 7 (T1), Week 13 (T2) and Week 52 (T3). Mean and SD will also be computed for the delta of CGI score between T1 and T0. Missing data will be replaced using multiple imputation methods, provided that the data is missing at random or completely at random.

Additionally, in order to take into account the difference between the features of both cohorts (RT and TAU), we will use a propensity score of being allocated to RT to adjust the different model. For the secondary criteria, these propensity scores will be applied to the other scales used for the assessment.

Costs will be estimated from the point of view of society for the follow up period (1 year) without discounting, due to the study duration. We will estimate in each group the median cost, mean and 95% confidence interval (95%CI) using bootstrap replications because of distribution skewedness and calculate the cost difference. Health-related QOL is collected using the EQ-5D-5 L self-administered questionnaire at baseline, at 3 months and at 1 year. The utility values will be based on French tariffs. The efficacy of the intervention will be estimated by quality adjusted life years (QALYs), using the EQ-5D-5 L scores and the time between treatment sessions. The difference in QALYs is the difference in the area between the utility curves for the two groups. Between-group comparisons of QALYs and costs will be performed with the appropriate statistical tests for their distribution, with a significance threshold of .05 (two-sided test).

The incremental cost utility ratio is the difference in average total costs divided by the difference in average total QALYs. The joint comparison of costs and effects will use nonparametric bootstrapping with 1000 resamples and generate the scatterplot on the cost effectiveness plane as a well as an acceptability curve.

### Sample size

This study aims to assess the usefulness of RI. The number of patients should be considered as a continuous variable, which contributes to the precision of the estimates of the results: –Means, course secondary to treatments, based on anterior studies using the delta (Day 1 – week 7) on the PCL-S score. One should expect a pre−/post-treatment PCL-S difference of 30 points with a SD of 15–25 points [7]. If a symptom reduction of 5–9 points on the PCL-S represents a favorable response and a reduction greater than 10 represents a clinically meaningful reduction, one should expect that 70% of patients display a favorable response to the RT treatment [7]. Based on those results, the confidence intervals will evolve according to the plot proposed below. For example, a 95%CI calculated over 200 patients will vary between 27 and 33.

## Discussion

The fight against terrorism should involve all mental health specialties. To fulfill that role, effective and innovating multimodal treatments are required. At its conclusion, Paris MEM will become the largest pragmatic trial to date assessing whether RT works under the usual healthcare practice that prevails in the aftermath of a large-scale man-made disaster (effectiveness). Paris MEM aims to determine if RT can become the preferred first-line therapeutic intervention for traumatic stress following a wide scale catastrophic event, considering that it is easy to teach to clinical staff and may treat trauma-related disorders using a lesser amount of time and resources than TAU (efficiency). RT could reinforce the therapeutic arsenal for the treatment of PTSD patients, not only for communities in western countries but also worldwide for terror- or disaster-stricken communities who wish to undo some of the psychological damage they sustained.

One of the aims of the Paris MEM study is to provide an educational phase on an innovative therapeutic method able to help practitioners of the various healthcare institutions of Paris to treat PTSD. While building the experimental design, one of our main concerns was the feasibility and acceptability of the treatment procedure from the therapists’ and patients’ perspectives. As mentioned in the Methods section, one of the main outcomes will combine cost and effectiveness 1 year following the treatment. In that sense, we aim for a non-inferiority procedure compared to TAU, hoping for a better cost-utility outcome of the RT method.

The absence of randomization could be considered a limitation. However, this choice is in line with the choice of a pragmatic trial, and ensures feasibility, considering that –based on our prior experience- we expect that patients will express a preference for RT compared to TAU. Due to the extraordinary circumstances in which this study was initiated, we felt that it would be wiser to conduct the trial by asking the study participants to express their preference toward the treatment they wished to receive (RT or TAU). Ultimately, the choice of treatment of the participants will inform us as to the treatment acceptability of RI. Additionally, recruitment would be easier using a free choice approach. As mentioned previously, a regression model highlighting the course of PTSD symptom severity over time will allow us to measure the proportion of patients responding to treatment and achieving remission. The comparison of the two treated cohorts will involve a propensity score of choosing RT treatment, in order to take into account measurable confusion factors and compensate for the absence of randomization. Also, to limit selection bias, eligibility criteria are the same for the two groups, except for contra-indications to propranolol. In the context of the terrorist attacks and ensuing mass suffering, and given the existence of efficacious treatments (SSRIs or psychotherapies), this effectiveness study is not compatible with the implementation of a placebo arm. A placebo controlled trial was recently published and showed the efficacy of RT versus placebo [7].

The absence of blinding is another limitation in our study. Neither the patients nor the assessors are blind for the primary criterion of assessment (PCL-S score), as is always the case in psychotherapy research. A single-blind pre/post evaluation is conducted for the CGI criterion, with the same rater who does not participate in the patient’s care. In addition, because the main outcome is a PTSD symptom self-report –and not a clinician-based interview- there is no interviewer bias.

## Conclusion

Paris MEM was initiated in the context of the most severe attacks to occur in France since WWII. This study illustrates the importance given by the French health authorities and the AP-HP, to the consequences of acute stress in the context of terrorism. RT represents an innovative treatment that could prove to be the most cost-effective treatment proposed in this domain up to this day. We strongly believe that Paris MEM will increase our understanding of how to effectively alleviate post-traumatic suffering in the aftermath of mass traumatization, and that it has the potential to serve as an inspiring example to the world.

## Supplementary information

**Additional file 1.** Nine Dimensions for Assessing the Level of Pragmatism in a Trial, as Proposed in the Pragmatic–Explanatory Continuum Indicator Summary 2 (PRECIS-2) Tool (Ford I, Norrie J. 2016) [31].

## Data Availability

The datasets used and/or analyzed during the current study belong to the AP-HP (Assistance Publique - Hopitaux de Paris) research directorate and will be available by request to the corresponding author.

## Abbreviations

CBT: Cognitive-Behavioral Therapy
CGI: Clinical Global Impression
CHEERS: Consolidated Health Economic Evaluation Reporting Standards
EMDR: Eye Movement Desensitization and Reprocessing
EQ-5D-5 L: EuroQol Questionnaire
HAS: Haute Autorité de Santé or French Health Authority
HSCL-25: Hopkins Symptom Checklist
ITT: Intention to treat
MEDEC: MEDico-EConomic Questionnaire
MEDEC: Medico-Economic Questionnaire
MINI-S: Mini International Neuropsychiatric Interview for DSM-5
PCL-S: PTSD Checklist – Specific Version
PDEQ: Peritraumatic Dissociative Experience Questionnaire
PDI: Peritraumatic Distress Inventory
PTSD: Post traumatic disorder
QALYs: Quality adjusted life years
QFS: Social Functioning Questionnaire
RT: Reconsolidation therapy
TAU: Treatment as usual
WHOQOL-BREF: World Health Organization Quality of Life (brief)
WHOQOL-Bref: World Health Organization Quality of Life instrument
