# Supplementary material for: Paris MEM: a study protocol for an effectiveness and efficiency trial on the treatment of traumatic stress in France after the 2015–16 terrorist attacks
Source: BMC Psychiatry. 2019 Nov 8;19:351. doi: 10.1186/s12888-019-2283-4 (PMC6842179; doi:10.1186/s12888-019-2283-4)
Supplement: Supplementary file 1 [file 12888_2019_2283_MOESM1_ESM.docx]

Dimension	Assessment of Pragmatism
**Recruitment of investigators and participants**	
Eligibility	To what extent are the participants in the trial similar to patients who would receive this intervention if it was part of usual care?
Recruitment	How much extra effort is made to recruit participants over and above what would be used in the usual care setting to engage with patients?
Setting	How different are the settings of the trial from the usual care setting?
**The intervention and its delivery within the trial**	
Organization	How different are the resources, provider expertise, and organization of care delivery in the intervention group of the trial from those available in usual care?
Flexibility in delivery	How different is the flexibility in how the intervention is delivered from the flexibility anticipated in usual care?
Flexibility in adherence	How different is the flexibility in how participants are monitored and encouraged to adhere to the intervention from the flexibility anticipated in usual care?
**The nature of follow-up**	
Follow-up	How different is the intensity of measurement and the follow-up of participants in the trial from the typical follow-up in usual care?
**The nature, determination, and analysis of outcomes**	
Primary outcome	To what extent is the primary outcome of the trial directly relevant to participants?
Primary analysis	To what extent are all data included in the analysis of the primary outcome?
